# The neutrophil-to-C3 ratio: unveiling diagnostic efficacy for lupus nephritis and association with reduced retinal vascular density in systemic lupus erythematosus

**DOI:** 10.3389/fphar.2025.1484320

**Published:** 2025-02-19

**Authors:** Maierhaba Maitiyaer, Peiyi Li, Huangdong Li, Dingwen Jiang, Jingyu Zhang, Chuwei Yang, Xiaobin Yang, Chengmei Chen, Wenhui Huang, Zhiping Liu, Shuilian Yu

**Affiliations:** ^1^ Department of Rheumatology, The Second Affiliated Hospital of Guangzhou Medical University, Guangzhou, China; ^2^ Ophthalmic Center, The Second Affiliated Hospital of Guangzhou Medical University, Guangzhou, China; ^3^ Department of Clinical Medicine, The Second Clinical School of Guangzhou Medical University, Guangzhou, China; ^4^ E.N.T. Department, The Second Affiliated Hospital of Guangzhou Medical University, Guangzhou, China

**Keywords:** systemic lupus erythematosus, neutrophil-to-C3 ratio, lupus nephritis, retinal microvasculature, biomarker

## Abstract

**Background:**

Lupus nephritis (LN) frequently manifests as a significant complication in systemic lupus erythematosus (SLE) patients, with emerging research indicating a plausible correlation between subclinical retinal involvement and renal manifestations. This study aims to investigate the relationship between the neutrophil-to-C3 ratio (NC3R) and both LN as well as retinal microvasculature damage among SLE patients.

**Methods:**

In this cross-sectional study, a cohort of 220 participants (78 LN and 142 non-LN) was recruited. We assessed clinical indicators and organ involvement and conducted correlation analyses between NC3R and markers of lupus activity. Additionally, we analyzed the diagnostic performance of NC3R in diagnosing LN and constructed ROC curves. Variables such as clinical and laboratory data were screened by univariate and least absolute shrinkage and selection operator (LASSO) logistic regression modeling. After 10-fold cross-validation, the predictive model was built by multivariate logistic regression. We further examined the influence of NC3R on retinal vasculature density based on its cutoff value and conducted correlation analyses accordingly.

**Results:**

The LN group exhibited a significant increase in NC3R compared to the group without LN (5.9 vs. 4.5, *p* = 0.007). NC3R demonstrated positive correlations with 24-h proteinuria (R = 0.329, *p* < 0.001) and systemic lupus erythematosus disease activity index (SLEDAI) score (R = 0.268, *p* < 0.001). Multiple regression analysis revealed NC3R as an independent risk factor for LN (OR: 2.03, *p* = 0.025). NC3R was proven valuable in distinguishing LN patients (AUC: 0.613), with an optimal cutoff value of 6.40 (sensitivity: 48.1%, specificity: 72.0%). Our results indicated that the lower NC3R group (NC3R < 6.40) exhibited reduced vascular density, particularly within the macular region. Furthermore, we observed a positive correlation between NC3R levels and vascular density.

**Conclusion:**

NC3R demonstrated promising potential as a reliable indicator for predicting both LN and retinal microvasculature involvement. Consequently, the pre-treatment evaluation of NC3R had the potential to assist clinicians in identifying potential organ involvement among SLE patients.

## 1 Introduction

Systemic lupus erythematosus (SLE) is an autoimmune disease that triggers inflammation in multiple organs due to the loss of self-tolerance and the formation of autoantigens and immune complexes (Kiriakidou and Ching, 2020). Among the complications of SLE, lupus nephritis (LN) exhibits rapid progression, an unfavorable prognosis, and contributes significantly to morbidity and mortality (Hahn et al., 2012). Ocular involvement, including retinopathy, is prevalent in approximately one-third of SLE patients and plays a crucial role in assessing disease activity and prognosis, primarily due to active SLE retinal vasculopathy and reduced complement levels (Silpa-archa et al., 2016; Luboń et al., 2022).

Neutrophil immune dysregulation is implicated in SLE pathogenesis by activating type Ⅰ interferon, leading to the production of effector leukocytes, inflammatory mediators, and autoantibodies, which contribute to tissue damage affecting various organs, including the kidneys and eyes (Mistry et al., 2019). Complement system activation contributes to neutrophil recruitment and activation, with increased neutrophils in the kidneys associated with nephritis activity (Mohan and Putterman, 2015). Recent studies have identified a critical role for neutrophils in SLE disease progression, particularly in patients with lower complement levels and increased inflammatory cells such as neutrophils (Yu et al., 2019). The association between the neutrophil-to-C3 ratio (NC3R) and SLE disease activity has been shown, but its diagnostic value for identifying LN remains uncertain.

Identifying reliable biological markers for predicting renal and retinal vasculopathy in SLE patients is of utmost importance for effective monitoring and treatment. This study aims to investigate the predictive efficacy of NC3R in LN patients and its correlation with retinal microvascular damage, thereby assisting clinicians in early screening and prevention of organ damage.

## 2 Materials and methods

### 2.1 Participants

This study was a retrospective, single-center observational study and 220 patients with SLE were recruited from the Second Affiliated Hospital of Guangzhou Medical University between September 2019 and April 2024. All SLE patients were diagnosed according to the 2019 EULAR/ACR classification criteria for SLE (Aringer et al., 2019). The inclusion criteria for patients in the LN group were: (1) persistent proteinuria >0.5 g/day or >3+ by dipstick, and/or the presence of cellular casts (red cell, hemoglobin, granular, tubular, or mixed); or (2) renal biopsy demonstrating immune complex-mediated glomerulonephritis consistent with LN (Hahn et al., 2012). SLE disease activity was evaluated utilizing the systemic lupus erythematosus disease activity index (SLEDAI)-2K score (Gladman et al., 2002). The ocular inclusion criteria were defined as follows: (1) best-corrected visual acuity better than 0.1 log of the Minimum Angle of Resolution (LogMAR); (2) intraocular pressure <21 mmHg; (3) spherical equivalent < +2.5 D or >−6.0 D. Patients with infections, malignancies, and other inflammatory diseases were excluded for this study. The ocular exclusion criteria were defined as follows: (1) spherical equivalent > +6.0 D or <-6.0 D; (2) axial length ≥26 mm; (3) any ocular pathological changes detected on slit lamp, fundus color photography, or optical coherence tomography imaging; (4) history of previous ocular diseases such as glaucoma, cataract or ocular surgery, including refractive surgeries. Informed consent was obtained from all participants, and the study received approval from the Ethics Committee of the Second Affiliated Hospital of Guangzhou Medical University.

### 2.2 Rheumatological evaluation

Demographic data, clinical manifestations, and laboratory data were retrieved from patients’ medical records. Patient characteristics encompassed age, gender, disease duration, C-reactive protein (CRP), erythrocyte sedimentation rate (ESR), complement 3(C3), C4, neutrophil-to-lymphocyte ratio (NLR), NC3R. Kidney function was evaluated through the following biochemical assays: serum creatinine, glomerular filtration rate (GFR), and 24-h proteinuria. Additionally, we collected information regarding organ involvement in patients, including gastrointestinal involvement, neurological damage, arthritis, skin involvement, hematological abnormalities, and serositis. The NLR and NC3R are calculated by dividing the absolute neutrophil count by the absolute lymphocyte count or serum C3 value.

### 2.3 Ophthalmologic evaluation

**FIGURE 1:**
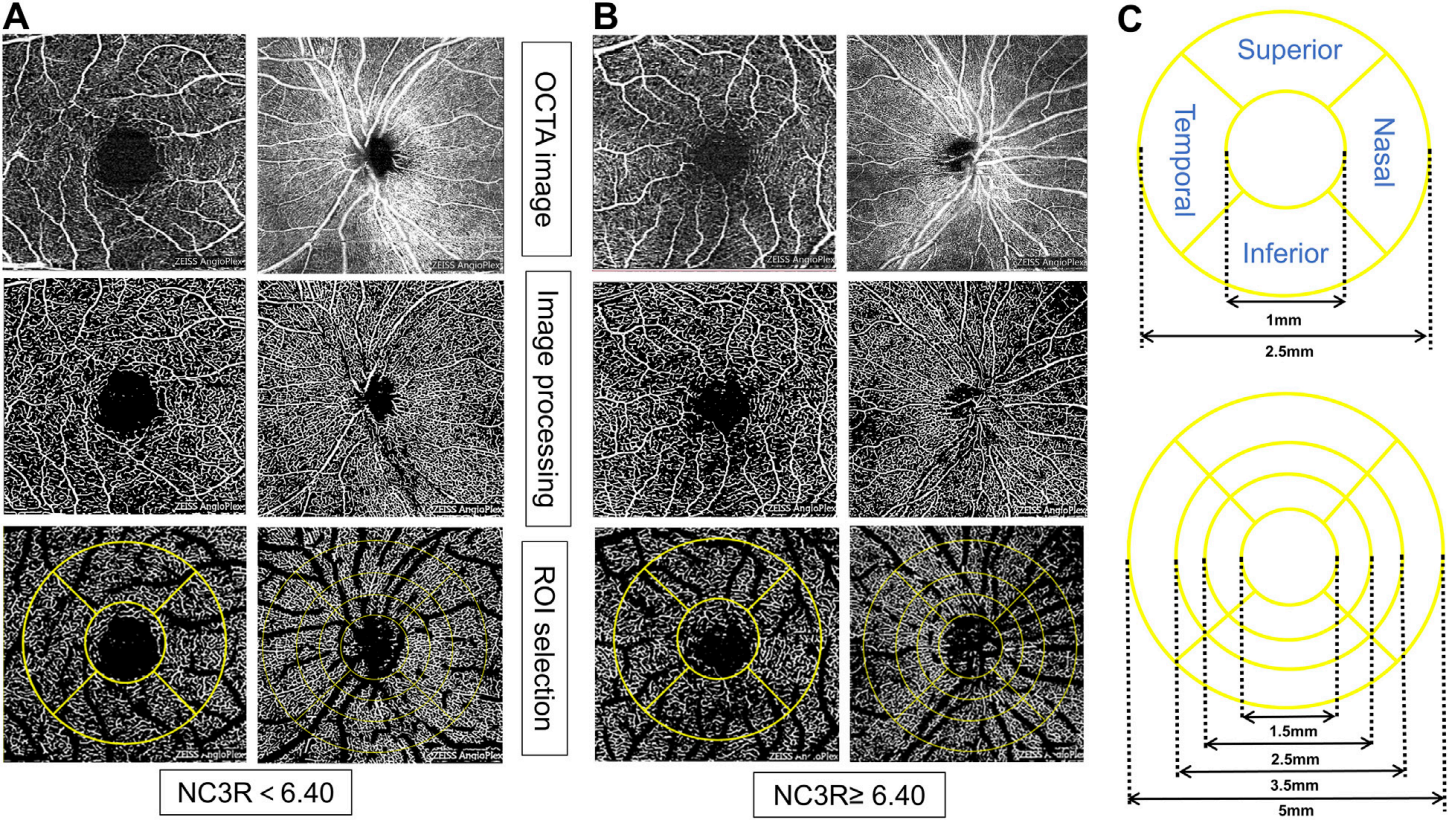
Representative OCTA images for measuring retinal microvasculature parameters in different NC3R groups. In **(A)**, the patient from the NC3R < 6.40 group exhibited a decreased vessel density, while in **(B)**, the patient from the NC3R ≥ 6.4 group displayed an increased vessel density. **(C)** showed the different circles and sides of macular and optic disc vascular density measured by OCTA. NC3R, neutrophil -to-C3 ratio; OCTA, Optical Coherence Tomography Angiography; ROI: region of interest.

According to the ETDRS (1991), a standard LogMAR chart was used to assess the best-corrected visual acuity (BCVA) in each eye, thereby evaluating central visual acuity. In the analysis of ocular vasculature, the region of interest (ROI) in the macular region was characterized by circular areas centered on the macula with diameters of 1 mm and 2.5 mm, respectively. The optic disc region was a circular zone centered on the optic disc with a diameter of 1.5 mm, 2.5 mm, 3.5 mm, and 5 mm, respectively. The parafoveal region referred to macular area 0.5–1.5 mm from the foveal center and the peripapillary region encompassed the optic nerve head. The ratio of flow pixels to total pixels was used to calculate macular vessel density (VD). The macular vessel length density (VLD) was calculated using the ratio of vessel length to total area. The foveal avascular zone (FAZ) area and perimeter were calculated by manually delineating the central avascular zone of the macula. The whole image referred to the entire 3 × 3 mm^2^ area centered on the macula/the entire 6 × 6 mm^2^ area centered on the optic disc. This circular region was subdivided further into superior, inferior, temporal, and nasal regions (Li et al., 2024) (Figures 1A–C).

### 2.4 Statistical analysis

SPSS software (version 25.0) and GraphPad Prism (version 10; GraphPad software) and Hiplot tools were used for analysis. The normality of distributions was tested by the Kolmogorov-Smirnov test. Data that followed a normal distribution were expressed as mean ± standard deviation and analyzed using Student’s t-test, while non-normally distributed data were presented as median and interquartile range (IQR) and evaluated with the Mann-Whitney *U*-test. The association between NC3R and relevant clinical indicators in SLE patients was assessed using Spearman’s correlation coefficient. The univariate logistic regression and multivariate logistic regression models examined if NLR and NC3R were associated with increased or decreased risk of LN in diagnosed patients. LASSO was applied to manage multiple predictors and reduce overfitting by selecting the most significant variables. A 10-fold cross-validation process was used to identify the optimal lambda value, based on the minimum binomial deviance. ROC and calibration curves evaluated the efficacy of the model. The maximum Youden index, calculated as sensitivity + specificity-1, from the ROC curve analysis, was used to determine the optimal cutoff values for NC3R and NLR. *p* < 0.05 constituted statistically significant results.

## 3 Result

### 3.1 Demographics and clinical parameters of SLE

**FIGURE 2:**
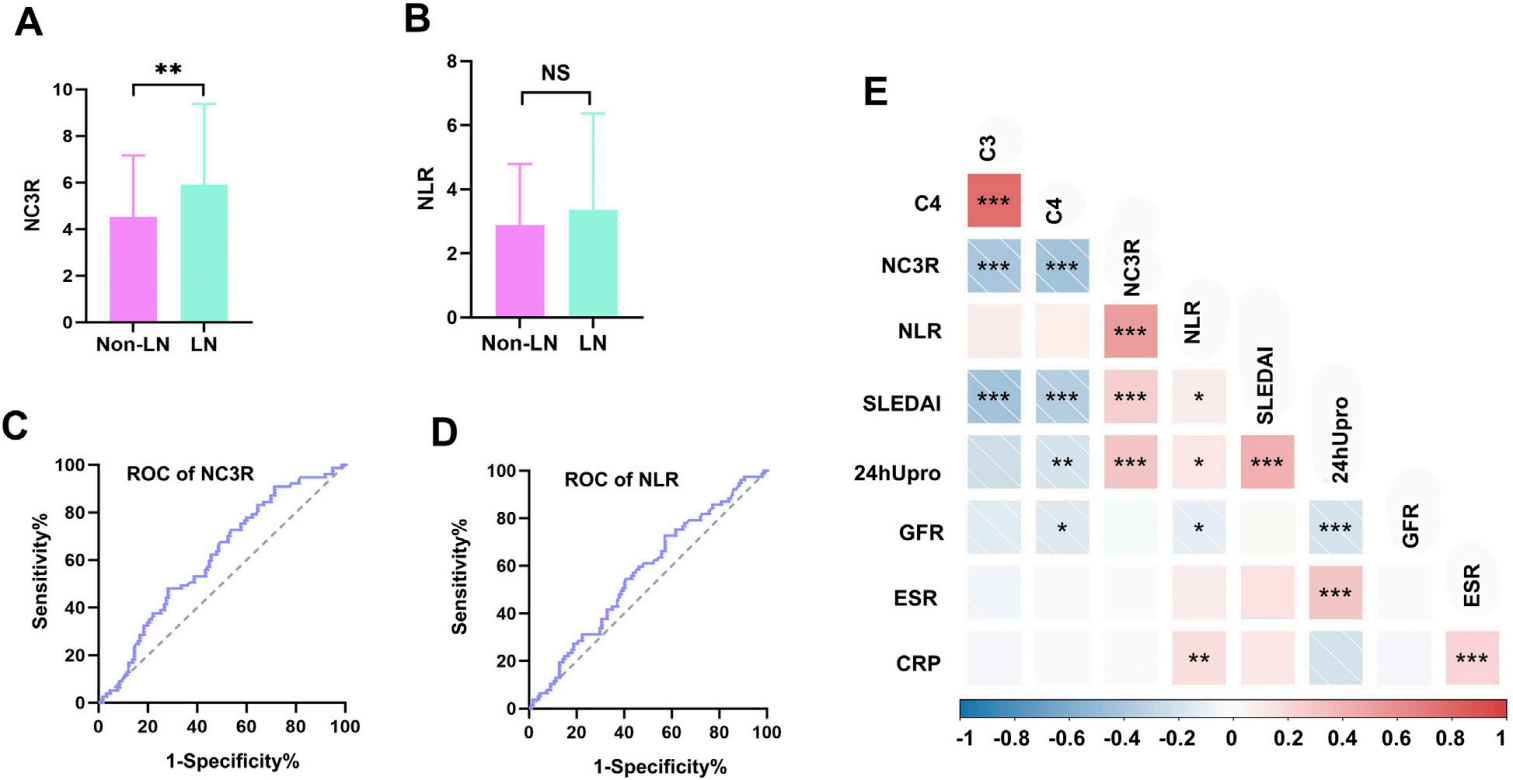
Comparison of NC3R and NLR in different groups, receiver operating characteristic (ROC) curve for predicting LN and correlations with clinical parameters. **(A, B)** The LN group showed a higher NC3R level compared to those without LN (5.9 vs. 4.5, P = 0.007), and no significant differences were observed in NLR between the two groups. **(C, D)** Area under curve (AUC) for NC3R and NLR are 0.613 (95% CI: 0.535-0.690), and 0.570 (95% CI: 0.491-0.649), respectively. **(E)** Correlations of NC3R, and NLR with clinical parameters in SLE patients. LN, lupus nephritis; NLR, neutrophil-to-lymphocyte ratio; NC3R, neutrophil -to-C3 ratio; 24 h Upro, 24-h proteinuria; CRP, C-reactive protein; ESR,erythrocyte sedimentation rate; C3, complement 3; C4, complement 4; GFR, glomerular filtration rate. *means P < 0.05; ** means P < 0.01; *** means P < 0.001.

A total of 220 Chinese SLE patients were recruited, including 206 women, and 14 men. They were divided into two groups based on the diagnosis of LN, 142 patients without LN and 78 patients with LN. Demographic and clinical characteristics were summarized in Table 1. The mean disease duration was no significant difference between the two groups. Participants with LN showed significantly higher levels of SLEDAI score (12.5 vs. 10.0, *p* = 0.017). Notably, LN patients showed a higher NC3R level compared to those without LN (5.9 vs. 4.5, *p* = 0.007) (Figure 2A). However, there were no significant differences observed in NLR between the two groups (Figure 2B). Serologically, LN patients had a significantly lower GFR, creatinine, and albumin level, and a higher 24-h proteinuria level (all *p* < 0.01). Gastrointestinal involvement was significantly higher in LN patients compared to non-LN patients (11.5% vs. 2.1%, p = 0.005). However, there were no significant differences in other organ involvements, including neurological damage, APS, arthritis, skin involvement, hematological damage, and serositis (Table 1). A total of 47 patients with LN underwent renal biopsy, including 2 with class II, 4 with class III, 2 with class III + V, 13 with class IV, 21 with class IV + V, and 5 with class V (Table 1). We further investigated the immunotherapy treatment in both groups. The results showed that the proportions of patients using prednisolone, hydroxychloroquine, and cyclosporine were similar between the two groups. However, a higher proportion of patients in the LN group received tacrolimus (7.7% vs. 0.7%, *p* = 0.009) and mycophenolate mofetil treatment (65.4% vs. 12.7%, *p* < 0.001). In contrast, a greater percentage of patients without LN were treated with methotrexate (11.3% vs. 2.6%, *p* = 0.024).

**TABLE 1:** Clinical characteristics and laboratory results of the patients with and without LN.

Variables	Non-LN (n = 142)	LN (n = 78)	P
Demographic characteristics			
Age (years, mean ± s.d.)	40.0 ± 15.8	36.3 ± 13.6	0.080
Gender (Male/Female)	7/135	7/71	0.251
Clinical features			
SLE Duration (years)	3.0 (0.0,10.0)	4.0 (1.0, 10.0)	0.500
SLEDAI score	10.0 (7.0, 16.0)	12.5 (8.0, 17.5)	**0.017**
Serological features			
Anti-dsDNA positivity (%)	142 (100)	78 (100)	0.066
NLR	2.9 (2.0, 4.8)	3.4 (2.4, 6.3)	0.090
NC3R	4.5 (3.0, 7.1)	5.9 (3.8, 9.2)	**0.007**
ESR (<40), mm/h	29.0 (12.0, 48.0)	29.0 (13.0, 56.5)	0.347
CRP (≤6), mg/L	4.7 (0.8, 15.0)	3.1 (1.1, 12.2)	0.336
C3 (0.7–1.4),g/L	0.8 (0.6, 1.0)	0.7 (0.6, 1.0)	0.640
C4 (0.1–0.4), g/L	0.1 (0.1, 0.2)	0.1 (0.1, 0.2)	0.841
GFR (>90), ml/min	108.6 (93.0, 135.9)	102.4 (60.7, 122.1)	**0.005**
24 h Upro (0–150), mg/24 h	109.5 (72.0, 229.2)	661.5 (217.0, 2167.8)	**< 0.001**
Albumin (40–55), g/L	36.3 (32.4, 39.9)	33.0 (26.6, 36.5)	**< 0.001**
Creatinine (41–81), umol/L	57.5 (48.0, 67.2)	63.0 (52.5, 95.8)	**0.002**
Organ involvement,n (%)			
Gastrointestinal	3 (2.1)	9 (11.5)	**0.005**
Neurological	16 (11.3)	13 (16.7)	0.257
APS	18 (12.7)	6 (7.7)	0.257
Arthritis	40 (28.2)	19 (24.4)	0.542
Skin	19 (13.4)	10 (12.8)	0.907
Hematological	56 (39.4)	29 (37.2)	0.742
Serositis	10 (7)	4 (5.1)	0.775
Types of lupus nephritis			
Type II	NA	2	
Type III	NA	4	
Type III + V	NA	2	
Type IV	NA	13	
Type IV + V	NA	21	
Type V	NA	5	
Rheumatological treatment, n (%)			
Prednisolone	128 (90.1)	75 (96.2)	0.11
Hydroxychloroquine	125 (89.3)	70 (89.7)	0.916
Methotrexate	16 (11.3)	2 (2.6)	**0.024**
Mycophenolate mofetil	18 (12.7)	51 (65.4)	**<0.001**
Cyclosporin	8 (5.6)	3 (3.8)	0.75
Tacrolimus	1 (0.7)	6 (7.7)	**0.009**

Note: Values are median (interquartile range, IQR) unless stated otherwise; s.d., standard deviation; LN, lupus nephritis; Anti-dsDNA, anti-double strained deoxyribonucleic acid antibodies; SLEDAI, systemic lupus erythematosus disease activity index; NLR, neutrophil-to-lymphocyte ratio; NC3R, neutrophil -to-C3, ratio; ESR, erythrocyte sedimentation rate; CRP, C-reactive protein; C3, complement component 3; C4, complement component 4; GFR, glomerular filtration rate; 24 h proteinuria: 24-h proteinuria. APS: Antiphospholipid antibody syndrome. P values were calculated by the Mann-Whitney U test and X^2^ test. P values below 0.05 indicate statistical significance. The bold values represented results with statistical significance. Normal value ranges are given in parentheses after the indicator.

### 3.2 Comparison of NC3R levels among different types of LN

To further investigate the differences in NC3R levels among different types of LN, we compared NC3R levels across the various LN subtypes. Our results indicate that there were no statistically significant differences in NC3R levels between the groups (Supplementary Table S1).

### 3.3 ROC curves of NC3R and NLR for predicting LN

ROC curve analyses were conducted to compare the diagnostic performance of NC3R, and NLR. The AUC for NC3R was 0.613 (95% *CI*: 0.535-0.690), which was higher than the AUC for NLR 0.570 (95% CI: 0.491-0.649) (Figures 2C, D). The cutoff value of NC3R in identifying LN was 6.40, with a sensitivity of 48.1% and specificity of 72.0%. The cutoff value of NLR was 2.55, with a sensitivity of 72.7% and a specificity of 42.9% (Supplementary Table S2).

### 3.4 Correlations of NC3R, NLR with clinical parameters in SLE patients

We further investigated the relationship between NC3R, NLR, and various clinical parameters including 24-h proteinuria, GFR, C3, C4, ESR, CRP, and SLEDAI scores. NC3R exhibited a positive correlation with 24-h proteinuria (R = 0.329, *p* < 0.001) and SLEDAI score (R = 0.268, *p* < 0.001), but a negative association with C3 (R = −0.422, *p* < 0.001) and C4 (R = −0.408, *p* < 0.001). Similarly, we found that NLR exhibited a positive correlation with 24-h proteinuria (R = 0.148, *p* = 0.048), SLEDAI score (R = 0.152, *p* = 0.028), and CRP (R = 0.271, *p* = 0.002), but exhibited a negative correlation with GFR (R = −0.177, *p* = 0.012) as shown in Table 2; Figure 2E.

**TABLE 2:** Correlations of NC3R, NLR with clinical parameters in SLE patients.

Variables	NC3R		NLR	
	R	P	R	P
24 h Upro (mg/24 h)	0.329	**<0.001**	0.148	**0.048**
GFR (mL/min)	−0.095	0.178	−0.177	**0.012**
ESR (mm/h)	0.032	0.646	0.085	0.227
SLEDAI	0.268	**<0.001**	0.152	**0.028**
C3 (g/L)	−0.422	**<0.001**	0.023	0.740
C4 (g/L)	−0.408	**<0.001**	0.017	0.803
CRP (mg/L)	−0.014	0.844	0.217	**0.002**

Note: NC3R, neutrophil-to-C3, ratio; NLR, neutrophil-to-lymphocyte ratio; GFR, glomerular filtration rate; ESR, erythrocyte sedimentation rate; SLEDAI, systemic lupus erythematosus disease activity index; C3, complement component 3; C4, complement component 4; 24 h Upro, 24-h proteinuria. P values below 0.05 indicate statistical significance. The bold values represented results with statistical significance.

### 3.5 Univariate logistic analysis, LASSO regression and multivariate logistic analysis of independent factors associated with LN

**FIGURE 3:**
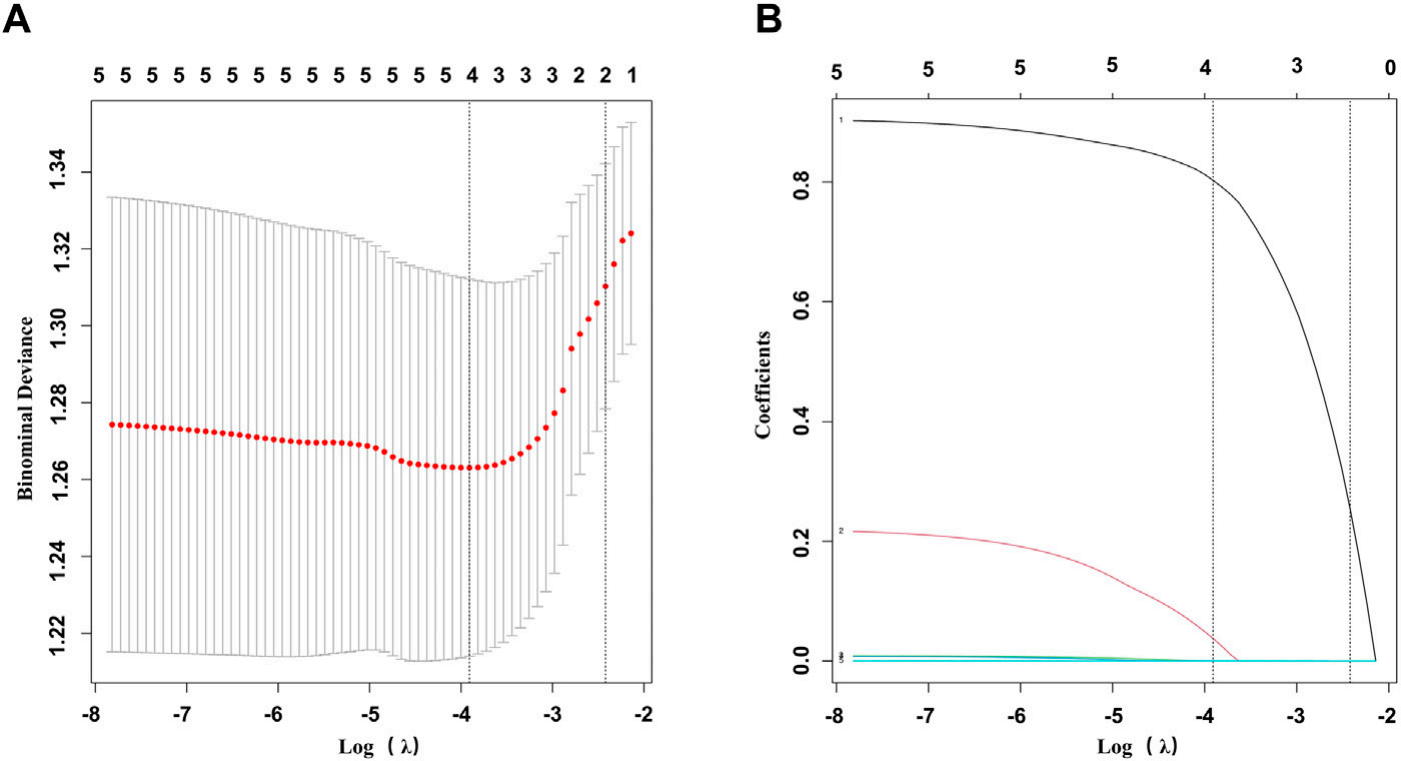
Feature selection was performed using LASSO binary logistic regression. **(A)** Filtering the best lambda by 10-fold cross-validation in the LASSO model. **(B)** LASSO model coefficient curves for candidate variables. LASSO, least absolute shrinkage and selection operator.

Univariate logistic analysis revealed possible variables correlated with LN. This was performed on the LN patients and non-LN patients. Results are presented in Table 3. NC3R and NLR were stratified based on a predefined cutoff value. Of the clinical features, NC3R ≥ 6.40 (OR = 2.37, *p* = 0.004), NLR ≥ 2.55 (OR = 2.01, *p* = 0.024), GFR (OR = 0.99, *p* = 0.005) and 24-h proteinuria (OR = 1, *p* < 0.001) were statistically significantly associated with LN. To avoid multicollinearity and overfitting, LASSO regression was applied following univariate logistic regression screening. A 10-fold cross-validation process identified the optimal lambda value (Figure 3A), with the minimum binomial deviance ensuring the best model performance (Figure 3B). Variables with non-zero coefficients, including NC3R (≥6.40) and NLR (≥2.55), were included in the final logistic regression model (Supplementary Table S3). Our findings revealed that NC3R (≥6.40) independently contributed to the risk of developing LN, with an odds ratio of 2.03 (95% CI: 1.09–3.78, *p* = 0.025).

**TABLE 3:** Univariate and multivariate logistic analysis of clinical parameters associated with LN.

Variables	Univariate logistic regression		Multivariate logistic regression	
	OR (95%CI)	P	OR (95%CI)	P
Disease Duration	0.99 (0.96∼1.03)	0.789		
Gender	1 (Ref)			
	0.53 (0.18∼1.58)	0.258		
NC3R < 6.40	1 (Ref)		1 (Ref)	
NC3R ≥ 6.40	2.37 (1.32∼4.27)	**0.004**	2.03 (1.09∼3.78)	**0.025**
NLR < 2.55	1 (Ref)		1 (Ref)	
NLR ≥ 2.55	2.01 (1.1∼3.68)	**0.024**	1.57 (0.82∼3.01)	0.171
ESR	1.01 (1∼1.01)	0.26		
CRP	0.99 (0.98∼1)	0.082		
GFR	0.99 (0.98∼1)	**0.005**		
C3	0.76 (0.29∼2.02)	0.588		
C4	1.23 (0.1∼14.76)	0.869		
24 h Upro	1 (1∼1)	**<0.001**		

Note: LN, lupus nephritis; NLR, neutrophil-to-lymphocyte ratio; NC3R, neutrophil-to-C3, ratio; OR: odds ratio; ESR, erythrocyte sedimentation rate; CRP, C-reactive protein; GFR, glomerular filtration rate; C3, complement component 3; C4, complement component 4; 24H Upro, 24-h proteinuria. A p-value below 0.05 indicates statistical significance. The bold values represented results with statistical significance.

### 3.6 Comparison of ophthalmic parameters among different NC3R groups

#### 3.6.1 Primary eye examination indexes

We conducted ophthalmological assessments on 52 patients and categorized them into two groups based on the NC3R cutoff value: 17 patients in the higher NC3R group (NC3R ≥ 6.40) and 35 patients in the lower NC3R group (NC3R < 6.40). No significant differences were observed in spherical equivalent (SEQ), BCVA (LogMAR), and intraocular pressure (IOP) between both groups of patients (*p* > 0.05, Table 4).

**TABLE 4:** Primary ocular examination parameters and comparison of macular and optic disc vascular density and vessel length density between different NC3R groups of patient.

Variables median (IQR)	NC3R < 6.4 (n = 35)	NC3R ≥ 6.4 (n = 17)	P
Ophthalmic conditions			
SEQ	−2.0 (−3.1, −0.7)	−2.3 (−4.0, −0.1)	0.924
LogMAR	−0.1 (−0.1, 0.0)	0.0 (−0.1, 0.0)	0.058
IOP(mmHg)	14.5 (11.8, 15.5)	14.5 (12.9, 15.0)	0.976
Microvascular VD and VLD			
Macula			
SCP parafoveal VD (%)			
1 mm Circle	20.9 (15.4, 23.4)	23.2 (22.2, 24.3)	**0.005**
2.5 mm Circle	36.1 (33.2, 37.8)	36.3 (35.4, 38.0)	0.246
Loop	39.3 (36.1, 40.9)	38.8 (37.7, 40.9)	0.592
Superior	39.2 (37.6, 42.0)	37.7 (36.8, 40.5)	0.441
Nasal	40.1 (36.9, 41.5)	40.0 (37.4, 41.7)	0.853
Inferior	38.5 (34.9, 40.4)	39.5 (37.8, 40.5)	0.116
Temporal	39.5 (35.9, 42.1)	40.4 (37.0, 41.3)	0.733
Whole image	37.6 (35.3, 39.0)	38.1 (36.8, 39.0)	0.441
SCP parafoveal VLD (%)			
1 mm Circle	3.6 (2.8, 4.1)	4.0 (3.8, 4.3)	**0.013**
2.5 mm Circle	6.3 (5.7, 6.6)	6.5 (6.0, 6.6)	0.354
Superior	6.9 (6.4, 7.2)	6.6 (6.2, 7.0)	0.552
Nasal	6.9 (6.2, 7.3)	7.0 (6.3, 7.3)	0.884
Inferior	6.7 (6.0, 7.1)	6.8 (6.5, 7.1)	0.513
Temporal	6.9 (6.3, 7.4)	7.0 (6.5, 7.2)	0.992
Whole image	6.6 (6.0, 6.9)	6.6 (6.3, 6.9)	0.565
DCP parafoveal VD (%)			
1 mm Circle	13.6 (11.2, 19.1)	18.7 (12.1, 22.3)	0.112
2.5 mm Circle	36.7 (35.1, 37.6)	38.5 (35.6, 39.4)	**0.028**
Loop	40.9 (39.4, 41.8)	42.3 (39.4, 43.0)	**0.036**
Superior	42.0 (39.0, 43.6)	42.2 (40.0, 43.9)	0.689
Nasal	39.7 (38.5, 42.7)	40.7 (38.0, 44.1)	0.675
Inferior	39.8 (37.6, 41.3)	42.0 (40.0, 42.6)	**0.009**
Temporal	40.9 (39.4, 42.8)	42.7 (40.8, 44.7)	0.062
Whole image	38.6 (37.2, 39.6)	39.8 (38.3, 40.9)	0.05
DCP parafoveal VLD (%)			
1 mm Circle	2.8 (2.2, 3.6)	3.6 (2.4, 4.4)	0.116
2.5 mm Circle	6.5 (6.3, 6.8)	6.9 (6.3, 7.1)	0.057
Superior	7.4 (7.0, 7.8)	7.5 (7.1, 7.9)	0.501
Nasal	7.1 (6.8, 7.4)	7.4 (6.7, 7.7)	0.592
Inferior	7.1 (6.7, 7.3)	7.5 (7.2, 7.7)	**0.015**
Temporal	7.3 (7.0, 7.5)	7.7 (7.1, 7.8)	0.131
Whole image	7.0 (6.8, 7.2)	7.2 (6.8, 7.4)	0.077
FAZ			
FAZ-area (mm^2^)	0.4 (0.3, 0.5)	0.3 (0.3, 0.4)	0.163
FAZ-circle (mm)	3.5 (3.1, 4.5)	3.3 (3.0, 4.5)	0.592
Optic disc			
SCP peripapillary VD (%)			
1.5 mm Circle	12.5 (8.9, 16.2)	12.2 (10.1, 14.7)	0.822
2.5 mm Circle	22.2 (18.7, 24.2)	22.3 (21.1, 23.5)	0.718
3.5 mm Circle	27.0 (24.1, 28.9)	27.6 (25.6, 28.3)	0.899
5.0 mm Circle	29.3 (26.8, 31.3)	29.9 (25.9, 31.5)	0.946
Inner Circle			
Superior	26.3 (21.6, 28.7)	23.3 (21.8, 27.3)	0.464
Nasal	28.2 (24.2, 32.9)	29.3 (23.6, 29.9)	0.578
Inferior	25.3 (19.6, 29.2)	27.8 (22.3, 30.2)	0.188
Temporal	27.9 (22.7, 33.4)	33.8 (25.3, 36.8)	0.181
Middle Circle			
Superior	31.2 (28.1, 34.2)	31.1 (28.0, 32.1)	0.513
Nasal	31.9 (27.6, 36.1)	32.0 (28.5, 34.8)	0.868
Inferior	30.2 (27.2, 32.7)	31.4 (27.8, 32.5)	0.689
Temporal	34.4 (32.2, 36.3)	35.1 (31.2, 39.8)	0.704
Outer Circle			
Superior	32.8 (29.6, 35.1)	32.4 (26.6, 34.4)	0.619
Nasal	31.9 (27.7, 34.4)	29.7 (26.9, 33.1)	0.396
Inferior	31.4 (29.3, 35.1)	31.7 (28.1, 34.7)	0.899
Temporal	32.5 (28.3, 35.3)	35.3 (30.1, 37.3)	0.126
Whole image	29.6 (27.5, 31.5)	30.2 (28.3, 32.5)	0.733
DCP peripapillary VD (%)			
1.5 mm Circle	18.5 (12.5, 25.3)	15.2 (12.3, 27.4)	0.946
2.5 mm Circle	19.9 (18.5, 21.1)	20.0 (18.3, 21.2)	0.977
3.5 mm Circle	22.8 (20.3, 24.1)	23.3 (20.8, 24.6)	0.592
5.0 mm Circle	24.2 (22.3, 26.0)	24.7 (23.4, 26.6)	0.441
Inner Circle			
Superior	20.7 (19.1, 24.3)	21.2 (18.5, 23.3)	0.868
Nasal	22.9 (19.4, 27.2)	22.7 (18.1, 26.8)	0.915
Inferior	22.5 (19.8, 25.5)	23.7 (18.6, 26.0)	0.43
Temporal	26.5 (23.3, 29.9)	27.6 (24.5, 30.9)	0.375
Middle Circle			
Superior	23.7 (22.0, 25.8)	25.4 (21.6, 26.7)	0.396
Nasal	28 .0 (23.8, 30.4)	27.3 (21.0, 31.6)	0.619
Inferior	25.8 (22.9, 29.3)	24.4 (22.1, 26.0)	0.375
Temporal	26.7 (23.2, 30.2)	28.9 (24.8, 29.7)	0.407
Outer Circle			
Superior	25.3 (22.6, 27.3)	26.0 (24.3, 28.2)	0.325
Nasal	29.5 (26.5, 31.9)	32.2 (24.2, 33.7)	0.501
Inferior	25.5 (22.3, 28.0)	25.3 (22.6, 26.9)	0.838
Temporal	24.5 (20.1, 28.7)	25.4 (22.9, 31.0)	0.501
Whole image	25.9 (23.0, 28.2)	25.2 (24.5, 27.9)	0.592

Note: NC3R, neutrophil -to-C3, ratio; OCTA, optical coherence tomography angiography; SEQ, spherical equivalent; LogMAR, logarithm of the minimum angle of resolution; IOP, intraocular pressure; SCP, superficial capillary plexus; DCP, deep capillary plexus; VD, vessel density; VLD, vessel length density; FAZ, foveal avascular zone; Parafoveal: the macular region located 0.5–1.5 mm from the foveal center; Peripapillary: optic disc region surrounding the optic nerve head. P values below 0.05 indicate statistical significance. The bold values represented results with statistical significance.

#### 3.6.2 Superficial and deep capillary density in different areas of the macula

The patients in the NC3R ≥ 6.40 group exhibited increased superficial capillary plexus vessel density (SCP-VD) and superficial capillary plexus vessel length density (SCP-VLD) in the 1 mm circle area (all *p* < 0.05). Additionally, an increase in deep capillary plexus vessel density (DCP-VD) in the 2.5 mm circle area, loop area, inferior area, and an increased deep capillary plexus vessel length density (DCP-VLD) in the inferior area were observed (all *p* < 0.05) (Table 4; Figures 1A, B).

#### 3.6.3 Foveal avascular zone parameters

A comparative analysis of FAZ parameters was undertaken between two groups of patients. Nevertheless, no statistically significant differences were identified in either FAZ area or FAZ circle measurements between them (all *p* > 0.05, Table 4).

#### 3.6.4 Superficial and deep capillary density in different areas of the optic disc

There were no significant differences observed in optic disc SCP-VD and DCP-VD in both groups (all *p* > 0.05, Table 4).

#### 3.6.5 Correlations of NC3R with microvascular vessel density in SLE patients

We conducted a correlation analysis between NC3R and retinal vascular density. Our findings indicated a positive correlation between NC3R and SCP-VD in the 1 mm circle area, 2.5 mm circle area, and SCP-VLD in the 1 mm circle area. In DCP-VD, there was a positive correlation with the 2.5 mm circle, loop area, inferior side, and temporal side, as well as with DCP-VLD in the 2.5 mm circle, inferior side, and temporal side. Notably, there is a negative correlation between NC3R and the FAZ area. (all *p* < 0.05) (Supplementary Table S4).

## 4 Discussion

Early diagnosis of LN is crucial due to its severe impact, with only 50%–70% of SLE patients achieving remission and 10%–20% progressing to end-stage renal disease within 5 years, highlighting its role in disease progression and adverse prognosis (Hahn et al., 2012). Lupus retinopathy primarily manifests as immune complex-mediated microangiopathy, indicating severe disease activity and its correlation with the central nervous system and renal involvement, making it a prognostic marker for poor survival outcomes (Palejwala et al., 2012; Gallagher et al., 2015). Conigliaro et al. found that SLE patients, especially those with kidney involvement, exhibited decreased retinal microvascular density compared to normal subjects (Conigliaro et al., 2018; Conigliaro et al., 2019). Additionally, Ushiyama et al. observed higher levels of serum creatinine in SLE patients with retinopathy compared to those without retinopathy (Ushiyama et al., 2000).

Complement system activation, complement fragment production, and deposition, as well as subsequent inflammation, are integral to the pathogenesis of SLE. Moreover, the release of compounds and antimicrobial peptides from neutrophils in SLE patients contributes to tissue and organ damage and inflammation (Kaplan, 2011). Previous reports have indicated lower complement levels and higher inflammatory cell counts in SLE patients compared to healthy individuals (Charlesworth et al., 1989; Bosch, 2011; Fresneda Alarcon et al., 2021). Recent research has shed light on the fact that many components involved in NETosis function as autoantigens in autoimmune processes (Knight et al., 2012). Studies have also indicated that remnants of neutrophil activation, such as NET structures, can be deposited in glomeruli even in the absence of intact neutrophils within the kidneys (Frangou et al., 2019). In patients with LN, significantly elevated levels of autoantibodies are found in their serum, exceeding concentrations in the kidneys by a factor of 10^3^. Moreover, target antigens have been detected in the NETosis matrix, providing support for the underlying concept that anti-podocyte and anti-implanted antigen antibodies form in circulation and subsequently accumulate within the glomeruli (Bonanni et al., 2015). Therefore, this study aimed to explore the association between NC3R, and LN.

Previous studies have established NLR as a valuable marker for assessing disease activity in various conditions such as rheumatoid arthritis, Bechet disease, psoriatic arthritis, and adult-onset Still’s disease (Kim et al., 2016; Avci et al., 2017; Seo et al., 2017; Erre et al., 2019). Similarly, Liu et al., Tang et al., and Firizal et al. have reported NLR potential utility in monitoring disease activity and kidney damage in SLE patients (Firizal et al., 2020; Liu et al., 2020; Tang et al., 2022). Given the pivotal role of C3 in immune complex deposition and organ damage in SLE, particularly in LN, we chose NC3R for its ability to offer more specific insights into the disease mechanisms and provide a comprehensive understanding of both immune dysregulation and inflammation. Notably, our analysis revealed that NC3R had superior predictive capabilities in differentiating LN patients compared to NLR. Consistent with the findings of Yu et al. (2019), who reported its utility as an inflammatory marker for evaluating disease activity in SLE, we observed significantly higher levels of NC3R in LN patients. Furthermore, our study demonstrated a positive association between NC3R and 24-h proteinuria, a crucial indicator for diagnosing LN. Through multivariate logistic regression analysis, we determined that NC3R remained an independent risk factor for poor renal outcomes, even after adjusting for other variables (OR: 2.03, P = 0.035). In addition, our ROC analysis indicated that NC3R, not NLR, could serve as an additional diagnostic tool for identifying LN, displaying higher specificity compared to NLR (72.0% vs 42.9%). These findings suggested that NC3R may serve as an indicator of renal involvement in SLE patients.

Lupus retinopathy is characterized by microangiopathy, severe vascular occlusion, and vasculitis (Silpa-archa et al., 2016; Luboń et al., 2022). The vasculitis involves the deposition of immune complexes on endothelial cells, initiating complement activation and heightened phagocytosis, subsequently leading to the release of additional inflammatory mediators (Giorgi et al., 1999). Notably, our results indicated that the lower NC3R group (NC3R < 6.40) showed reduced vascular density, particularly pronounced in the macular retina. Lower NC3R levels may reflect increased complement activation, particularly C3, suggesting ongoing inflammation in small vessels (Sun et al., 2013), including those in the retina. Furthermore, we found a positive correlation between vascular density and NC3R levels, suggesting that NC3R may serve as a potential marker for vascular inflammation and complement activation in SLE retinopathy. Consequently, we hypothesize that the reduced vascular density in SLE patients is attributable to complement-mediated vasculitis, and NC3R may indicate potential retinal damage.

This study has several limitations. First, the cross-sectional design restricts our ability to fully explore the role of NC3R in LN. Second, further longitudinal studies are necessary to validate these findings. Third, the absence of a normal control group, along with potential influences from therapeutic medications, SLE itself, and coexisting conditions, may have affected NC3R levels. Despite these limitations, we observed significant correlations between NC3R, LN, and ocular involvement in SLE patients.

## 5 Conclusion

In conclusion, LN patients exhibited higher levels of NC3R compared to those without LN, with a positive correlation with 24-h proteinuria. NC3R emerged as a risk factor for LN and displayed significant diagnostic utility in identifying LN. Remarkably, patients in the lower NC3R group displayed a decreased retinal vessel density. These findings underscore the potential of NC3R as a predictive marker for LN and for evaluating retinal microvasculature vessel density in SLE patients.

## Data Availability

The raw data supporting the conclusions of this article will be made available by the authors, without undue reservation.
