# Supplementary material for: The neutrophil-to-C3 ratio: unveiling diagnostic efficacy for lupus nephritis and association with reduced retinal vascular density in systemic lupus erythematosus
Source: Front Pharmacol. 2025 Feb 19;16:1484320. doi: 10.3389/fphar.2025.1484320 (PMC11880234; doi:10.3389/fphar.2025.1484320)
Supplement: Supplementary file 1 [file Table1.docx]

| Variables | Total  (n = 47) | II  (n = 2) | III  (n = 4) | III+V (n = 2) | IV  (n = 13) | IV+V (n = 21) | V  (n = 5) | p |
| --- | --- | --- | --- | --- | --- | --- | --- | --- |
| NC3R, Median (IQR) | 6.0 (3.9, 9.1) | 4.4 (4.3, 4.5) | 4.4 (3.8, 5.4) | 5.0 (4.3, 5.7) | 6.3 (4.9, 11.1) | 6.7 (3.8, 9.2) | 6.5 (4.1, 7.5) | 0.599 |

**Supplementary Table S1**:Comparison of NC3R levels among different types of LN patients.

Notes:NC3R:neutrophil-to-C3 ratio. P values below 0.05 indicate statistical significance
