# Supplementary material for: The neutrophil-to-C3 ratio: unveiling diagnostic efficacy for lupus nephritis and association with reduced retinal vascular density in systemic lupus erythematosus
Source: Front Pharmacol. 2025 Feb 19;16:1484320. doi: 10.3389/fphar.2025.1484320 (PMC11880234; doi:10.3389/fphar.2025.1484320)
Supplement: Supplementary file 2 [file Table2.docx]

**Supplementary Table S2. Statistical evaluation of NLR, NC3R in predicting LN patients**

| ROC Curve  Item | Cutoff  value | AUC | 95%CI | Sensitivity (%) | Specificity (%) |
| --- | --- | --- | --- | --- | --- |
| NLR | 2.55 | 0.570 | 0.491 ~ 0.649 | 72.7 | 42.9 |
| NC3R | 6.40 | 0.613 | 0.535~0.690 | 48.1 | 72.0 |

Note:LN, lupus nephritis; NLR, neutrophil-to-lymphocyte ratio; NC3R, neutrophil-to-C3 ratio; 24h proteinuria: 24-hour proteinuria; ROC: Receiver operating characteristic; AUC: Area under curve, P values below 0.05 indicates statistical significance.
