# Supplementary material for: The neutrophil-to-C3 ratio: unveiling diagnostic efficacy for lupus nephritis and association with reduced retinal vascular density in systemic lupus erythematosus
Source: Front Pharmacol. 2025 Feb 19;16:1484320. doi: 10.3389/fphar.2025.1484320 (PMC11880234; doi:10.3389/fphar.2025.1484320)
Supplement: Supplementary file 3 [file Table3.docx]

**Supplementary Table S3. Coefficient estimates from LASSO regression for clinical variables.**

| Variables | mean squared  error coefficient | standard error of the  minimum distance coefficient |
| --- | --- | --- |
| NC3R(≥6.40) | 0.803 | 0.254 |
| NLR(≥2.55) | 0.038 | 0 |
| CRP | 0 | 0 |
| GFR | 0 | 0 |
| 24hUpro | 0 | 0 |

Note: NC3R, neutrophil-to-C3 ratio; NLR, neutrophil-to-lymphocyte ratio; CRP, C-reactive protein; GFR, glomerular filtration rate; 24hUpro: 24-hour proteinuria.
