# Supplementary material for: The neutrophil-to-C3 ratio: unveiling diagnostic efficacy for lupus nephritis and association with reduced retinal vascular density in systemic lupus erythematosus
Source: Front Pharmacol. 2025 Feb 19;16:1484320. doi: 10.3389/fphar.2025.1484320 (PMC11880234; doi:10.3389/fphar.2025.1484320)
Supplement: Supplementary file 4 [file Table4.docx]

**Supplementary Table S4:Correlation of microvascular vessel density with NC3R**

| **Variables** | **NC3R** | |
| --- | --- | --- |
|  | **R** | **P** |
| **Macula** |  |  |
| **SCP parafoveal VD(%)** |  |  |
| 1mm Circle | 0.432 | **0.001** |
| 2.5mm Circle | 0.286 | **0.040** |
| Loop | 0.209 | 0.138 |
| Superior | 0.055 | 0.700 |
| Nasal | 0.010 | 0.945 |
| Inferior | 0.249 | 0.075 |
| Temporal | 0.240 | 0.087 |
| Whole image | 0.207 | 0.140 |
| **SCP parafoveal VLD(%)** |  |  |
| 1mm Circle | 0.407 | **0.003** |
| 2.5mm Circle | 0.263 | 0.059 |
| Superior | 0.069 | 0.625 |
| Nasal | 0.046 | 0.746 |
| Inferior | 0.158 | 0.264 |
| Temporal | 0.241 | 0.085 |
| Whole image | 0.180 | 0.202 |
| **DCP parafoveal VD(%)** |  |  |
| 1mm Circle | 0.156 | 0.271 |
| 2.5mm Circle | 0.328 | **0.018** |
| Loop | 0.350 | **0.011** |
| Superior | 0.227 | 0.106 |
| Nasal | -0.096 | 0.500 |
| Inferior | 0.366 | **0.008** |
| Temporal | 0.411 | **0.002** |
| Whole image | 0.266 | 0.057 |
| **DCP parafoveal VLD(%)** |  |  |
| 1mm Circle | 0.126 | 0.372 |
| 2.5mm Circle | 0.286 | **0.040** |
| Superior | 0.246 | 0.078 |
| Nasal | -0.013 | 0.930 |
| Inferior | 0.312 | **0.024** |
| Temporal | 0.343 | **0.013** |
| Whole image | 0.260 | 0.063 |
| **FAZ** |  |  |
| FAZ-area(mm^2^) | -0.285 | **0.040** |
| FAZ-circle(mm) | -0.115 | 0.416 |
| **Optic disc** |  |  |
| **SCP peripapillary VD(%)** |  |  |
| 1.5mm Circle | -0.089 | 0.532 |
| 2.5mm Circle | -0.025 | 0.859 |
| 3.5mm Circle | -0.084 | 0.553 |
| 5.0mm Circle | -0.082 | 0.563 |
| Inner Circle |  |  |
| Superior | -0.168 | 0.234 |
| Nasal | -0.136 | 0.338 |
| Inferior | 0.153 | 0.280 |
| Temporal | 0.164 | 0.247 |
| Middle Circle |  |  |
| Superior | -0.134 | 0.345 |
| Nasal | -0.050 | 0.723 |
| Inferior | -0.053 | 0.708 |
| Temporal | -0.014 | 0.919 |
| Outer Circle |  |  |
| Superior | -0.161 | 0.254 |
| Nasal | -0.145 | 0.306 |
| Inferior | -0.132 | 0.349 |
| Temporal | 0.165 | 0.241 |
| Whole image | 0.009 | 0.947 |
| **DCP peripapillary VD(%)** |  |  |
| 1.5mm Circle | 0.135 | 0.340 |
| 2.5mm Circle | -0.103 | 0.467 |
| 3.5mm Circle | -0.020 | 0.887 |
| 5.0mm Circle | 0.017 | 0.907 |
| Inner Circle |  |  |
| Superior | -0.185 | 0.190 |
| Nasal | -0.040 | 0.776 |
| Inferior | 0.109 | 0.440 |
| Temporal | 0.056 | 0.695 |
| Middle Circle |  |  |
| Superior | -0.013 | 0.927 |
| Nasal | -0.092 | 0.518 |
| Inferior | -0.138 | 0.330 |
| Temporal | 0.091 | 0.520 |
| Outer Circle |  |  |
| Superior | -0.064 | 0.652 |
| Nasal | 0.095 | 0.504 |
| Inferior | -0.079 | 0.579 |
| Temporal | 0.063 | 0.659 |
| Whole image | 0.030 | 0.833 |

Note: NC3R: neutrophil -to-C3 ratio; OCTA, Optical Coherence Tomography Angiography; SCP: superficial capillary plexus; DCP: deep capillary plexus; VD: vessel density; VLD: vessel length density; FAZ: foveal avascular zone; Parafoveal: the macular region located 0.5–1.5 mm from the foveal center; Peripapillary: optic disc region surrounding the optic nerve head. P values below 0.05 indicate statistical significance. The bold values represented results with statistical significance.
